# The dynamics of COVID-19 with quarantined and isolation

**DOI:** 10.1186/s13662-020-02882-9

**Published:** 2020-08-14

**Authors:** Muhammad Altaf Khan, Abdon Atangana, Ebraheem Alzahrani, Fatmawati

**Affiliations:** 1 Informetrics Research Group, Ton Duc Thang University, Ho Chi Minh City, Vietnam; 2 Faculty of Mathematics and Statistics, Ton Duc Thang University, Ho Chi Minh City, Vietnam; 3 Faculty of Natural and Agricultural Sciences, University of the Free State, Bloemfontien, South Africa; 4 Department of Medical Research, China Medical University Hospital, Taichung, Taiwan; 5 Department of Mathematics, Faculty of Science, King Abdulaziz University, P. O. Box 80203, Jeddah, 21589 Saudi Arabia; 6 Department of Mathematics, Faculty of Science and Technology, Universitas Airlangga, 60115 Surabaya, Indonesia

**Keywords:** COVID-19 model, Quarantine and isolation, Fractal-fractional model, Estimation of the parameters, Numerical results

## Abstract

In the present paper, we formulate a new mathematical model for the dynamics of COVID-19 with quarantine and isolation. Initially, we provide a brief discussion on the model formulation and provide relevant mathematical results. Then, we consider the fractal-fractional derivative in Atangana–Baleanu sense, and we also generalize the model. The generalized model is used to obtain its stability results. We show that the model is locally asymptotically stable if $\mathcal{R}_{0}<1$. Further, we consider the real cases reported in China since January 11 till April 9, 2020. The reported cases have been used for obtaining the real parameters and the basic reproduction number for the given period, $\mathcal{R}_{0}\approx 6.6361$. The data of reported cases versus model for classical and fractal-factional order are presented. We show that the fractal-fractional order model provides the best fitting to the reported cases. The fractional mathematical model is solved by a novel numerical technique based on Newton approach, which is useful and reliable. A brief discussion on the graphical results using the novel numerical procedures are shown. Some key parameters that show significance in the disease elimination from the society are explored.

## Introduction

The coronavirus is a new, fatal and highly spreading infection that has put great panic around the globe since January 2, 2020. It is believed that the coronaviruses belong to a class of related viruses that initiate the diseases in birds and mammals. However, in humans, the coronaviruses initiate respiratory tract infections that can be insignificant, for example, the common cold. But others can be fatal, for instance, the SARS, MERS, and the new COVID-19. It is important to note that, although it is believed that they constitute a group of viruses, they can, however, be altered significantly, posing a risk factor. From the available literature, it is known that some of them can kill more than 30% of infected patients, for example, the MERS-Cov; nevertheless, other are really harmless, for example, the common cold. Up to date, the world has witnessed the appearance of seven strains of human coronaviruses, namely, Human coronavirus OC43 (HCoV-OC43), human coronavirus 229E (HCoV-229E), severe acute respiratory syndrome coronavirus (SARS-CoV), human coronavirus NL63 (HCoV-NL63), human coronavirus HKU1, Middle East respiratory syndrome-related coronavirus (MERS-CoV), and finally the latest version, called 2019-nCoV.

In general, it is known that the coronavirus can initiate direct or indirect viral or bacterial pneumonia, respectively. In this paper, we are interested more in the latest version of the so-called 2019-nCoV, which is also believed to be originated from bats. However, there are many controversies around its origin. If one assumes that such a virus is originated from bats, the first question one would ask is if such bats are new to our world, and if not, why such a virus has not spread before? Does this mean that such a virus has not been in contact with humans before? It was believed that the virus may have come in contact with humans, white humans began to eat bats without being properly cooked. However, if this hypothesis is correct and gives the mode of transmission of such a virus, one would go back to some villages in Africa where villagers directly eat fruits that were previously bitten by these bats. Also, in some of those villages, the bats can be consumed, killed, cleaned, and cooked, it is therefore possible that during the process of cleaning, villagers are exposed to the virus if really such a virus is received from bats. These observations make it suspicious to believe that the latest virus is originated from bats. On the other hand, there exist several books that were written in 1981, for instance, the Eyes of Darkness, where the author gives a clear narrative on how and where the breakout of the virus will start. In another book, titled “The End of the World Book”, the author gives a clear date when this pandemic will take place. It has become a trend that the attention of humans has shifted toward sport, music, and other social activities, production of knowledge does not matter anymore, scientists do not really have a say in their various societies. From the narrator of the book “The Eyes of Darkness”, it is believed that the virus is a biological weapon.

There are number of mathematical models that reported the COVID-19 dynamics, see [1]. In [1], a mathematical model for Wuhan outbreak has been presented with real statistical cases. The authors provide detailed analysis of the infection based on the real data. A mathematical model for COVID-19 to predict its dynamics for Italy is proposed in [2]. In another study, the authors studied the dynamics of COVID-19 in Italy [3]. A fractional model for intercity network is considered in [4]. A mathematical model of COVID-19 and its simulations are considered in [5]. A model of COVID-19 using fractional derivative has been considered in [6]. Recently, a coronavirus model has been considered mathematically in [7], where the authors used the real data from Pakistan and explored the possible control of infection and its elimination from Pakistan. The data of Ghana and its analysis through a mathematical model have been considered in [8], where the possible elimination of the virus from the country has been studied. In another study, the author explored the dynamics of coronavirus with the lockdown effect, where comprehensive statistical and mathematical results were explored for a better understanding of the infection [9].

While the aim of this paper is not agreeing or disagreeing with the discussion underpinning the origin of this virus, we shall recall that mathematicians use mathematical models to understand, control, and predict the spread of a given infectious disease. They use mathematical tools called differential operators to construct systems of mathematical equations that are able to replicate the real world scenario. Very recently, Atangana and Altaf [6] suggested a novel mathematical model able to predict the number of susceptible, infected, dead, recovered, and other individuals. Their mathematical model suggested a reproductive number of $\mathcal{R}_{0}=2.4829$, a value that is in good agreement with that suggested by the WHO. The mathematical model predicted an exponential increase in infections and deaths, which indeed is in good agreement with the real world observation. Nevertheless, in their model, the effects of temperature, distancing, and source of infection were not included.

Mathematical models that addressed the physical or biological problems are numerous in the literature; see, for example, [10–20]. For instance, the authors in [10] considered a numerical scheme to obtain the solution of a fractional optimal-control problem. Whereas the authors in [11] presented results for a fractional optimal-control problem with a general derivative. In [12], the authors considered a nonsingular operator and obtained the results for fractional Euler–Lagrange equations. The dynamics of human liver with Caputo–Fabrizio derivative has been studied in [13]. The time fractional optimal-control problem with nonsingular operator has been discussed in [14]. A fractional model for HRSV with optimal control has been analyzed in [15]. The authors studied the fish model with Mittag-Leffler law in [16]. Using the new method, called Bernstein wavelets, to obtain the solution of SIR model was considered in [17]. In [18], the authors studied the exothermic reactions model with Mittag-Leffler law. The solution of a cold plasma problem with hybrid method was studied in [19]. A new fractional model for measles with vaccine application was considered in [20].

We extend the model given in [6] by incorporating the quarantine and isolations classes to predict the dynamics of COVID-19 in China with real data. The model formulation is shown initially using integer order and then the model is generalized to obtain the fractal-fractional model. The fractional models and their applications to biological and physical problems are numerous in the literature; see [21–25]. We provided above comprehensive details on the mathematical modeling of the coronavirus infection and its background results. We organized the rest of the work in this paper as follows: The model formulation is shown in Sect. 2. Some mathematical results for the model have been shown in Sect. 3. The basics of the fractal-fractional calculus and its application to the COVID-19 model are shown briefly in Sect. 4. In Sect. 5, we consider a new numerical approach for the solution of the fractional COVID-19 model with quarantine and isolation based on the Newton polynomial approach. Estimation of the model parameters is shown in Sect. 6. The numerical results are discussed briefly in Sect. 7 while the concluding remarks are shown in Sect. 8.

## Model formulation

### Formulation of coronavirus with quarantine and hospitalization

The disease dynamics of COVID-19 is now a global issue with millions of infections and deaths worldwide. The countries who restrict their individuals to isolation and quarantine get a decrease in the infection cases of COVID-19. The isolation and quarantine have been considered a useful control in order to get rid of this infection. Therefore, the model considered here is for the transmission dynamics of the novel coronavirus (2019-nCoV) with the analysis of the quarantine of exposed individuals and isolation of individuals infected with the disease clinically. We also considered in this study the asymptomatically infected individuals who take part in infection generation without any symptoms. Thus, the model total population $N(t)$ is divided into seven human subclasses, namely, the susceptible individuals $S(t)$, exposed $E(t)$ (infected, but not showing any disease symptoms), symptomatically infected or infected individuals $I(t)$ (with clinical symptoms), asymptomatically infected $A(t)$ (not showing any clinical symptoms), quarantined $Q(t)$, hospitalized $H(t)$, and the recovered individuals $R(t)$. The infection that is mainly caused due to the seafood market, which is considered here as $M(t)$, is an environment for generating the infection by visiting the market by the people for purchasing food. The assumptions above lead to the following system of evolutionary differential equations:

1 $$\begin{aligned} \begin{gathered} \frac{dS}{dt}=\varLambda -\mu S(t)-\lambda (t) S(t), \\ \frac{dE}{dt}=\lambda (t) S(t)- \bigl((1-\theta )\omega +\theta \rho + \mu +\delta _{1} \bigr)E(t), \\ \frac{dI}{dt}=(1-\theta )\omega E(t)-(\tau _{1}+\mu +\xi _{1}+ \gamma )I(t), \\ \frac{dA}{dt}=\theta \rho E(t)-(\tau _{2}+\mu )A(t), \\ \frac{dQ}{dt}=\delta _{1} E(t)-(\mu +\phi _{1}+\delta _{2}) Q(t), \\ \frac{dH}{dt}=\gamma I(t)+\delta _{2} Q(t)-(\mu +\phi _{2}+\xi _{2}) H(t), \\ \frac{dR}{dt}=\tau _{1} I(t)+\tau _{2}A(t)+ \phi _{1} Q(t)+\phi _{2} H(t)- \mu R(t), \\ \frac{dM}{dt}=q_{1} I(t)+q_{2} A(t)-q_{3} M(t), \end{gathered} \end{aligned}$$

where

2 $$\begin{aligned} \lambda (t)=\frac{\eta _{1} (I+\psi A)}{N}+\eta _{2} M. \end{aligned}$$

**Figure 1:**
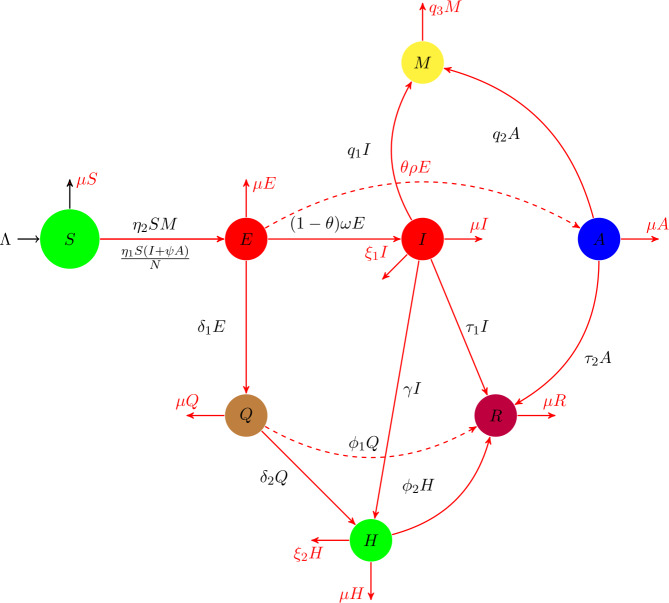
The description of the flow rate of the parameters of the model

Susceptible individuals acquire infection, following effective contacts with symptomatically infected, asymptomatically infected and the infection from the seafood market (*I*, *A*, *M*) shown by $\lambda (t)$. The birth rate for the susceptible individuals is given by *Λ*. The natural mortality rate of the human population is shown by *μ*. The healthy individuals require infection after contacting with infected and asymptomatically infected individuals by a rate $\eta _{1}$, while *ψ* denotes the transmissibility factor. The asymptomatic infection is generated by the parameter *θ*. The incubation periods are shown by *ω* and *ρ*. The parameters $\tau _{1}$, $\tau _{2}$, $\phi _{1}$, $\phi _{2}$ denote, respectively, the recovery of infected, asymptomatically infected, quarantined, and hospitalized individuals. The hospitalization rate of infected and quarantined individuals are shown respectively by *γ* and $\delta _{2}$. The disease death rate of infected and hospitalized individuals is shown by $\xi _{1}$ and $\xi _{2}$. The parameter $\delta _{1}$ represents the quarantine rate of exposed individuals. Individuals who are visiting the seafood market and catch the infection are increasing with rate $\eta _{2}$. The infection generated in the seafood market due to infected and asymptomatically infected is shown by the parameters $q_{1}$ and $q_{2}$, respectively, while the removal of infection from the market is given by $q_{3}$. The above transfer flow rate has been shown in Fig. 1.

## Model analysis

### Solution positivity

#### Lemma 1

*Let the initial data be* $G(0)\ge 0$, *where* $G(t)=(S(t), E(t), I(t), A(t), Q(t), H(t), R(t), M(t))$. *Then*, *for every* $t>0$, *we have nonnegative solution for model* (1). *Further*,

$$\begin{aligned} \lim_{t\rightarrow \infty } N(t)\leq \frac{\varLambda }{\mu }, \end{aligned}$$

*with* $N(t)=S(t)+ E(t)+ I(t)+ A(t)+Q(t)+H(t)+R(t)$.

#### Proof

Consider $t_{1}=\sup \{t>0: G(t)>0\}$. So, $t_{1}>0$. It follows from the first equation of system (1) that

3 $$\begin{aligned} \frac{d S}{dt} =& \varLambda -\mu S(t)-\lambda (t) S(t), \end{aligned}$$

with $\lambda (t)=\frac{\eta _{1} (I+\psi A)}{N}+\eta _{2} M$. Then, we can write equation (3) as

4 $$\begin{aligned} \frac{d}{dt} \biggl\{ S(t)~\exp \biggl( \mu t+ \int ^{t_{1}}_{0}\lambda ( \rho )\,d\rho \biggr) \biggr\} =\varLambda \exp \biggl( \mu t+ \int ^{t_{1}}_{0} \lambda (\rho )\,d\rho \biggr). \end{aligned}$$

Hence,

5 $$\begin{aligned} S(t_{1})~\exp \biggl( \mu t_{1}+ \int ^{t_{1}}_{0}\lambda (\rho )\,d\rho \biggr)-S(0)= \varLambda \exp \biggl( \mu x+ \int ^{x}_{0}\lambda (\zeta )\,d\zeta \biggr)\,dx, \end{aligned}$$

so that

6 $$\begin{aligned} S(t_{1}) =&S(0)\exp \biggl\{ - \biggl(\mu t_{1}+ \int ^{t_{1}}_{0}\lambda ( \rho )\,d\rho \biggr) \biggr\} +\exp \biggl\{ - \biggl(\mu t_{1}+ \int ^{t_{1}}_{0} \lambda (\rho )\,d\rho \biggr) \biggr\} \\ & {}\times \int ^{t_{1}}_{0} \varLambda \exp \biggl( \mu x+ \int ^{x}_{0} \lambda (\zeta )\,d\zeta \biggr)\,dx>0. \end{aligned}$$

For the rest of the equations, we can take a similar approach as above for system (1) to show $G(t)>0$ for every $t>0$. To show the other claim, note that $0< S(0)\leq N(t)$, $0< E(0)\leq N(t)$, $0< I(0)\leq N(t)$, $0< A(0)\leq N(t)$, $0< Q(0) \leq N(t)$, $0< H(0)\leq N(t)$, $0\leq R(0)\leq N(t)$. Adding all the equations of system (1) except for the last equation, we have

$$\begin{aligned} \frac{dN}{dt}=\varLambda -\mu N-\xi _{1} I-\xi _{2} H\leq \varLambda -\mu N, \end{aligned}$$

so

$$\begin{aligned} \lim_{t\rightarrow \infty } N(t)\leq \frac{\varLambda }{\mu }. \end{aligned}$$

 □

Next, we show the invariant regions for the given model (1). Consider the feasible region *Ω*, given by

$$\begin{aligned} \varOmega = \biggl\{ \bigl(S(t), E(t), I(t) ,A(t) , Q(t) , H(t), R(t)\bigr)\in \mathbb{R}^{7}_{+}:N(t)\leq \frac{\varLambda }{\mu }, M(t) \in \mathbb{R}_{+}: \frac{\varLambda }{\mu } \frac{q_{1}+q_{2}}{q_{3}} \biggr\} . \end{aligned}$$

We have the following results for this feasible region.

#### Lemma 2

*The region given by* *Ω* *is positively invariant for model* (1) *with the nonnegative initial conditions in* (7).

#### Proof

Adding the components of human population in model (1), we have

$$\begin{aligned} \frac{dN}{dt}=\varLambda -\mu N-\xi _{1} I-\xi _{2} H\leq \varLambda -\mu N. \end{aligned}$$

Hence, $\frac{dN(t)}{dt}\leq 0$, if $N(0)\ge \frac{\varLambda }{\mu }$. So, $N(t)\leq N(0)e^{-\mu t}+\frac{\varLambda }{\mu } (1-e^{-\mu t} )$. Thus, the region given by *Ω* is positively invariant. Also, if $N(0)>\frac{\varLambda }{\mu }$ and $N(0)>\frac{\varLambda }{\mu }$, then either the solution enters *Ω* in finite time, or $N(t)$ tends to $\frac{\varLambda }{\mu }$ asymptotically. So, the regions given by *Ω* attract all the solutions in $\mathbb{R}^{7}_{+}$. □

## A basic of fractal-fractional calculus and its application to the COVID-19 model

In this section, we discuss the essential literature related to the fractal-fractional operator and its applications to the model of COVID-19. The flowing definitions are taken from [26].

### Basic of fractal-fractional calculus

We present here some related results about the fractal-fractional operators.

#### Definition 1

For a function $g(t)\in W^{1}_{2} (0,1)$, $b>a$ and $\alpha _{1} \in [0, 1]$, the definition of Atangana–Baleanu derivative in the Caputo sense is given by

$$\begin{aligned} ^{ABC}_{0} D^{\alpha _{1}}_{t} g(t)= \frac{AB(\alpha _{1})}{1-\alpha _{1}} \int ^{t}_{0}\frac{d}{d\tau }g( \tau )E_{\alpha _{1}} \biggl[-\frac{\alpha _{1}}{1-\alpha _{1}}(t-\tau )^{ \alpha _{1}} \biggr]\,d\tau , \end{aligned}$$

where

$$\begin{aligned} AB(\alpha _{1})=1-\alpha _{1}+ \frac{\alpha _{1}}{\varGamma (\alpha _{1})}. \end{aligned}$$

#### Definition 2

Suppose that $g(t)$ is continuous on an open interval $(a, b)$, then the fractal-fractional integral of $g(t)$ of order $\alpha _{1}$ having Mittag-Leffler-type kernel and given by

$$\begin{aligned} ^{\mathrm{FFM}} J^{\alpha _{1}, \alpha _{2}}_{0, t}\bigl(g(t)\bigr)= \frac{\alpha _{1} \alpha _{2}}{AB(\alpha _{1})\varGamma (\alpha _{1})} \int ^{t}_{0} s^{ \alpha _{2}-1} g(s) (t-s)^{\alpha _{1}}\,ds+ \frac{\alpha _{2}(1-\alpha _{1})t^{\alpha _{2}-1} g(t)}{AB(\alpha _{1})}. \end{aligned}$$

### A fractional COVID-19 model

We present the dynamics of the COVID-19 model (1) using fractal-fractional Atangana–Baleanu derivative. We have the following model:

7 $$\begin{aligned} \begin{gathered} ^{FF}D^{\alpha _{1}, \alpha _{2}}_{0, t} S = \varLambda -\mu S(t)- \lambda (t) S(t), \\ ^{FF}D^{\alpha _{1}, \alpha _{2}}_{0, t} E = \lambda (t) S(t)- \bigl((1- \theta )\omega +\theta \rho +\mu +\delta _{1} \bigr)E(t), \\ ^{FF}D^{\alpha _{1}, \alpha _{2}}_{0, t} I = (1-\theta )\omega E(t)-( \tau _{1}+\mu +\xi _{1}+\gamma )I(t), \\ ^{FF}D^{\alpha _{1}, \alpha _{2}}_{0, t} A = \theta \rho E(t)-(\tau _{2}+ \mu )A(t), \\ ^{FF}D^{\alpha _{1}, \alpha _{2}}_{0, t} Q = \delta _{1} E(t)-(\mu + \phi _{1}+\delta _{2}) Q(t), \\ ^{FF}D^{\alpha _{1}, \alpha _{2}}_{0, t} H = \gamma I(t)+\delta _{2} Q(t)-( \mu +\phi _{2}+\xi _{2}) H(t), \\ ^{FF}D^{\alpha _{1}, \alpha _{2}}_{0, t}R = \tau _{1} I(t)+\tau _{2}A(t)+ \phi _{1} Q(t)+\phi _{2} H(t)-\mu R(t), \\ ^{FF}D^{\alpha _{1}, \alpha _{2}}_{0, t} M = q_{1} I(t)+q_{2} A(t)-q_{3} M(t), \end{gathered} \end{aligned}$$

where

8 $$\begin{aligned} \lambda (t)=\frac{\eta _{1} (I+\psi A)}{N}+\eta _{2} M, \end{aligned}$$

and $\alpha _{1}$ and $\alpha _{2}$ respectively represent fractal and fractional order.

The initial conditions are

9 $$\begin{aligned} \begin{gathered} S(0)=S_{0}\geq 0,\qquad E(0)=E_{0}\geq 0,\qquad I(0)=I_{0}\geq 0,\qquad A(0)=A_{0} \geq 0, \\ Q(0)=Q_{0}\geq 0,\qquad H(0)=H_{0}\geq 0,\qquad R(0)=R_{0}\geq 0,\qquad M(0)=M_{0} \geq 0. \end{gathered} \end{aligned}$$

### Stability analysis

We show the analysis of model (7) in this subsection. The disease-free equilibrium of the model (1) is given by $P_{0}$ and obtained as follows:

$$\begin{aligned} P_{0}= \bigl\{ S^{0}, 0,0,0,0,0,0,0 \bigr\} = \biggl\{ \frac{\varLambda }{\mu }, 0,0,0,0,0,0,0 \biggr\} . \end{aligned}$$

The basic reproduction number for model (7) using the next generation approach [27] is shown below:

$$\begin{aligned}& F= \begin{pmatrix} 0 & \eta _{1} & \psi \eta _{1} & 0 & 0 & \frac{\varLambda \eta _{2}}{\mu } \\ 0 & 0 & 0 & 0 & 0 & 0 \\ 0 & 0 & 0 & 0 & 0 & 0 \\ 0 & 0 & 0 & 0 & 0 & 0 \\ 0 & 0 & 0 & 0 & 0 & 0 \\ 0 & 0 & 0 & 0 & 0 & 0 \end{pmatrix},\\& V= \begin{pmatrix} k_{1} & 0 & 0 & 0 & 0 & 0 \\ (\theta -1) \omega & k_{2} & 0 & 0 & 0 & 0 \\ -\theta \rho & 0 & k_{3} & 0 & 0 & 0 \\ -\delta _{1} & 0 & 0 & k_{4} & 0 & 0 \\ 0 & -\gamma & 0 & -\delta _{2} & k_{5} & 0 \\ 0 & -q_{1} & -q_{2} & 0 & 0 & q_{3} \end{pmatrix}, \\& \mathcal{R}_{0}= \biggl( \frac{\theta k_{2} \rho (\eta _{2} \varLambda q_{2}+\eta _{1} \mu q_{3} \psi )+(1-\theta ) k_{3} \omega (\eta _{2} \varLambda q_{1}+\eta _{1} \mu q_{3} )}{k_{1} k_{2} k_{3} \mu q_{3}} \biggr) \\& \hphantom{\mathcal{R}_{0}}= \underbrace{\frac{\eta _{1} \theta \rho \psi }{k_{1} k_{3}}}_{ \mathcal{R}_{1}}+ \underbrace{ \frac{\eta _{1} (1-\theta ) \omega }{k_{1} k_{2}}}_{\mathcal{R}_{2}}+ \underbrace{\frac{\eta _{2} \theta \varLambda \rho q_{2}}{k_{1} k_{3} \mu q_{3}}}_{ \mathcal{R}_{3}}+ \underbrace{\frac{\eta _{2} (1-\theta ) \varLambda q_{1} \omega }{k_{1} k_{2} \mu q_{3}}}_{ \mathcal{R}_{4}}, \end{aligned}$$

where $k_{1}=\delta _{1}+\theta \rho +(1-\theta ) \omega +\mu $, $k_{2}=\gamma +\mu +\xi _{1}+\tau _{1}$, $k_{3}=\mu +\tau _{2}$, $k_{4}=\delta _{2}+\mu +\phi _{1}$, and $k_{5}=\mu +\xi _{2}+\phi _{2}$. In the following, we show the local stability of model (7).

#### Theorem 1

*System* (1) *at equilibrium point* $P_{0}$*is locally asymptotically stable if* $\mathcal{R}_{0}<1$.

#### Proof

Calculating the Jacobian matrix of system (7) at $P_{0}$, we get

10 $$\begin{aligned} J_{P_{0}}= \begin{pmatrix} -\mu & 0 & -\eta _{1} & -\psi \eta _{1} & 0 & 0 & 0 & - \frac{\varLambda \eta _{2}}{\mu } \\ 0 & -k_{1} & \eta _{1} & \psi \eta _{1} & 0 & 0 & 0 & \frac{\varLambda \eta _{2}}{\mu } \\ 0 & (1-\theta ) \omega & -k_{2} & 0 & 0 & 0 & 0 & 0 \\ 0 & \theta \rho & 0 & -k_{3} & 0 & 0 & 0 & 0 \\ 0 & \delta _{1} & 0 & 0 & -k_{4} & 0 & 0 & 0 \\ 0 & 0 & \gamma & 0 & \delta _{2} & -k_{5} & 0 & 0 \\ 0 & 0 & \tau _{1} & \tau _{2} & \phi _{1} & \phi _{2} & -\mu & 0 \\ 0 & 0 & q_{1} & q_{2} & 0 & 0 & 0 & -q_{3} \end{pmatrix}. \end{aligned}$$

It can be seen from the above Jacobian matrix $J_{P_{0}}$ that the eigenvalues −*μ*, −*μ*, $-k_{4}$, $-k_{5}$ have negative real parts. There are more eigenvalues (four) that can be obtained through the equation given by

11 $$\begin{aligned} \lambda ^{4}+c_{1}\lambda ^{3}+c_{2}\lambda ^{2}+c_{3} \lambda +c_{4}=0, \end{aligned}$$

where

12 $$\begin{aligned} \begin{gathered} c_{1} = k_{1}+k_{2}+k_{3}+q_{3}, \\ c_{2} = k_{1} k_{3} (1- \mathcal{R}_{1} )+k_{1} k_{2} (1- \mathcal{R}_{2} )+ (k_{1}+k_{2}+k_{3} ) q_{3}+k_{2} k_{3}, \\ c_{3} = k_{1} k_{2} k_{3} (1- \mathcal{R}_{2} )+k_{1} k_{3} q_{3} (1-\mathcal{R}_{3} )+k_{1} k_{2} q_{3} (1- \mathcal{R}_{4} ) \\ \hphantom{c_{3} =}{} + \underbrace{k_{2} k_{3} q_{3}-\eta _{1} \bigl(\theta k_{2} \rho \psi +q_{3} \bigl(\theta \rho \psi +(1-\theta ) \omega \bigr) \bigr)}, \\ c_{4} = k_{1} k_{2} k_{3} q_{3} (1-\mathcal{R}_{0}). \end{gathered} \end{aligned}$$

Obviously, the coefficients $c_{i}$ for $i=1, 2, 3, 4 $ given above are positive and the last one is positive whenever $\mathcal{R}_{0}<1$. Further, it will easily satisfy the Rough–Hurtwiz criterion $c_{1} c_{2} c_{3}-c^{2}_{1} c_{4} -c^{2}_{3}>0$. The Rough–Hurtwiz conditions can be satisfied simply, which will ensure the stability of model (7) at the disease-free point $P_{0}$, which is locally asymptotically stable if $\mathcal{R}_{0}<1$. □

Next, we obtain the equilibria at the endemic point, $P_{1}= \{S^{*}, E^{*}, I^{*}, A^{*}, Q^{*}, H^{*}, R^{*}, M^{*} \}$, given by

13 $$\begin{aligned} \textstyle\begin{cases} S^{*}=\frac{\varLambda ^{*} }{\lambda +\mu } , \\ E^{*}=\frac{\lambda ^{*} S^{*}}{k_{1}}, \\ I^{*}=\frac{ (1-\theta ) \omega E^{*}}{k_{2}}, \\ A^{*}=\frac{ \theta \rho E^{*}}{k_{3}}, \\ Q^{*}=\frac{\delta _{1} E^{*}}{k_{4}}, \\ H^{*}=\frac{\gamma I^{*}+\delta _{2} Q}{k_{5}}, \\ R^{*}= \frac{ \tau _{2}A^{*}+ \phi _{2} H^{*}+ \tau _{1} I^{*}+ \phi _{1} Q^{*}}{\mu }, \\ M^{*}=\frac{ q_{2} A^{*} + q_{1}I^{*}}{q_{3}}. \end{cases}\displaystyle \end{aligned}$$

Inserting the above result into

14 $$\begin{aligned} \lambda (t)=\frac{\eta _{1} (I+\psi A)}{N}+\eta _{2} M, \end{aligned}$$

we have

$$\begin{aligned} F\bigl(\lambda ^{*}\bigr)=l_{1}\bigl(\lambda ^{*}\bigr)^{2}+l_{2}\lambda ^{*}+l_{3}, \end{aligned}$$

where

$$\begin{aligned} l_{1} =&k_{1} k_{2} k_{3} q_{3} \bigl(k_{3} \bigl(\delta _{1} k_{2} \bigl(\delta _{2} (\mu +\phi _{2} )+k_{5} (\mu + \phi _{1} ) \bigr)+k_{4} k_{6} \bigr)+\theta k_{2} k_{4} k_{5} \rho (\mu +\tau _{2} ) \bigr), \\ l_{2} =&k_{1} k_{2} k_{3} \mu q_{3} \bigl(k_{3} \bigl(\delta _{1} k_{2} \bigl(\delta _{2} (\mu +\phi _{2} )+k_{5} (\mu + \phi _{1} ) \bigr)+k_{4} k_{8} \bigr)+\theta k_{2} k_{4} k_{5} \rho (-\eta _{1} \psi +\mu +\tau _{2} ) \bigr) \\ &{}+\eta _{2} k_{7} \varLambda \bigl((1-\theta ) k_{3} q_{1} \omega - \theta k_{2} \rho q_{2} \bigr)+k_{1}^{2} k_{2}^{2} k_{4} k_{5} k_{3}^{2} \mu q_{3}, \\ l_{3} =&k_{1}^{2} k_{2}^{2} k_{3}^{2} k_{4} k_{5} \mu ^{2} q_{3}(1- \mathcal{R}_{0}), \end{aligned}$$

and

$$\begin{aligned} k_{6} =&\gamma (1-\theta ) \omega (\mu +\phi _{2} )+k_{5} \bigl((1-\theta ) \omega (\mu +\tau _{1} )+k_{2} \mu \bigr), \\ k_{7} =&k_{3} \bigl(-\delta _{1} k_{2} \bigl(\delta _{2} ( \mu +\phi _{2} )+k_{5} (\mu +\phi _{1} ) \bigr)-k_{4} k_{6} \bigr)-\theta k_{2} k_{4} k_{5} \rho (\mu +\tau _{2} ), \\ k_{8} =&\gamma (1-\theta ) \omega (\mu +\phi _{2} )+k_{5} \bigl((1-\theta ) \omega (-\eta _{1}+\mu +\tau _{1} )+k_{2} \mu \bigr). \end{aligned}$$

Here, $l_{1}>0$, and $l_{3}$ depends on the sign of $\mathcal{R}_{0}$, which is positive when $\mathcal{R}_{0}<1$ and negative when $\mathcal{R}_{0}>1$. We summarize the above as follows:

#### Theorem 2

*System* (7) *has the following properties*:

(i) *If* $l_{3}<0$*and* $\mathcal{R}_{0}>1$, *then there exists a unique endemic equilibrium*;
(ii) *If* $l_{2}<0$*and* $l_{3}=0$, *then we have a unique endemic equilibrium*;
(iii) *If* $l_{3}>0$, $l_{2}<0$*and their discriminant is positive then two endemic equilibria exist*; *and*
(iv) *No possibilities of equilibria otherwise*.

It can be seen from the first point (i) of Theorem (2) that for $\mathcal{R}_{0}>1$, we have clearly a unique positive endemic equilibrium. Theorem (2)(iii) gives the possibility of backward bifurcation when $\mathcal{R}_{0}<1$.

## A new numerical procedure

In order to present the numerical algorithm for the fractal-fractional COVID-19 model (7), we first describe the general system and present the steps by considering the Cauchy problem below:

15 $$\begin{aligned} {}^{{\mathrm{FFM}}}_{0}D^{\alpha _{1}, \alpha _{2}}_{ t} x(t)=g \bigl(t, x(t)\bigr). \end{aligned}$$

The following is obtained by integrating the above equation:

16 $$\begin{aligned} x(t)-x(0) =&\frac{1-\alpha _{1}}{C(\alpha _{1})}\alpha _{2} t^{\alpha _{2}-1}g\bigl(t, x(t)\bigr) \\ &{}+ \frac{\alpha _{1}\alpha _{2}}{C(\alpha _{1})\varGamma (\alpha _{1})} \int ^{t}_{0} \tau ^{\alpha _{2}-1}g\bigl(\tau , x(\tau )\bigr) (t-\tau )^{ \alpha _{1}-1}\,d\tau . \end{aligned}$$

Let $K(t, x(t))=\alpha _{2}t^{\alpha _{2}-1}g(t, x(t))$, then equation (16) becomes

17 $$\begin{aligned} x(t)-x(0)=\frac{1-\alpha _{1}}{C(\alpha _{1})}K \bigl(t, x(t)\bigr)+ \frac{\alpha _{1}}{C(\alpha _{1})\varGamma (\alpha _{1})} \int ^{t}_{0} K\bigl( \tau , x(\tau )\bigr) (t- \tau )^{\alpha _{1}-1}\,d\tau . \end{aligned}$$

At $t_{n+1}=(n+1)\Delta t$, we have

18 $$\begin{aligned} x(t_{n+1})-x(0) =&\frac{1-\alpha _{1}}{C(\alpha _{1})}K \bigl(t_{n}, x(t_{n})\bigr) \\ &{}+ \frac{\alpha _{1}}{C(\alpha _{1})\varGamma (\alpha _{1})} \int ^{t_{n+1}}_{0} K\bigl(\tau , x(\tau )\bigr) (t_{n+1}-\tau )^{\alpha _{1}-1}\,d\tau . \end{aligned}$$

Also, we have

19 $$\begin{aligned} x(t_{n+1}) =&x(0)+\frac{1-\alpha _{1}}{C(\alpha _{1})}K \bigl(t_{n}, x(t_{n})\bigr) \\ &{}+ \frac{\alpha _{1}}{C(\alpha _{1})\varGamma (\alpha _{1})}\sum ^{n}_{j=2} \int ^{t_{j+1}}_{t_{j}} K\bigl(\tau , x(\tau )\bigr) (t_{n+1}-\tau )^{\alpha _{1}-1}\,d \tau . \end{aligned}$$

Approximating the function $K(t, x(t))$, using the Newton polynomial, we have

20 $$\begin{aligned} P_{n}(\tau ) =&K\bigl(t_{n-2}, x(t_{n-2}) \bigr)+ \frac{K(t_{n-1}, x(t_{n-1}) )-K(t_{n-2}, x(t_{n-2}) )}{\Delta t}(\tau -t_{n-2}) \\ & {}+ \frac{K(t_{n}, x(t_{n}) )-2 K(t_{n-1}, x(t_{n-1}) )+K(t_{n-2}, x(t_{n-2}) )}{2 (\Delta t)^{2}}(\tau -{t_{n-2}}) (\tau -{t_{n-1}}). \end{aligned}$$

Inserting equation (20) into (19), we have

21 $$\begin{aligned} x^{n+1} =&x^{0}+\frac{1-\alpha _{1}}{C(\alpha _{1})}K \bigl(t_{n}, x(t_{n})\bigr) \\ &{}+\frac{\alpha _{1}}{C(\alpha _{1})\varGamma (\alpha _{1})}\sum^{n}_{j=2} \int ^{t_{j+1}}_{t_{j}} \biggl\{ K\bigl(t_{j-2}, x^{j-2}\bigr)+ \frac{K(t_{j-1}, x^{j-1} )-K(t_{j-2}, x^{j-2})}{\Delta t}(\tau -t_{j-2}) \\ & {}+ \frac{K(t_{j}, x^{j} )-2 K(t_{j-1}, x^{j-1} )+K(t_{j-2}, x^{j-2}) }{2 (\Delta t)^{2}}(\tau -{t_{j-2}}) ( \tau -{t_{j-1}}) \biggr\} \\ &{}\times (t_{n+1}-\tau )^{\alpha _{1}-1}\,d\tau . \end{aligned}$$

Reordering the above equation, we have

22 $$\begin{aligned} x^{n+1} =&x^{0}+\frac{1-\alpha _{1}}{C(\alpha _{1})}K \bigl(t_{n}, x(t_{n})\bigr) \\ &{}+\frac{\alpha _{1}}{C(\alpha _{1})\varGamma (\alpha _{1})}\sum^{n}_{j=2} \biggl[ \int ^{t_{j+1}}_{t_{j}} K\bigl(t_{j-2}, x^{j-2} \bigr) (t_{n+1}-\tau )^{ \alpha _{1}-1}\,d\tau \\ &{}+ \int ^{t_{j+1}}_{t_{j}} \frac{K(t_{j-1}, x^{j-1} )-K(t_{j-2}, x^{j-2})}{\Delta t}(\tau -t_{j-2}) (t_{n+1}-\tau )^{\alpha _{1}-1}\,d \tau \\ & {}+ \int ^{t_{j+1}}_{t_{j}} \frac{K(t_{j}, x^{j} )-2 K(t_{j-1}, x^{j-1} )+K(t_{j-2}, x^{j-2} )}{2 (\Delta t)^{2}}(\tau -{t_{j-2}}) (\tau -{t_{j-1}}) \\ &{}\times (t_{n+1}-\tau )^{\alpha _{1}-1}\,d\tau \biggr]. \end{aligned}$$

Writing further equation (22), we have

23 $$\begin{aligned} x^{n+1} =&x^{0}+\frac{1-\alpha _{1}}{C(\alpha _{1})}K \bigl(t_{n}, x(t_{n})\bigr) \\ &{}+\frac{\alpha _{1}}{C(\alpha _{1})\varGamma (\alpha _{1})}\sum^{n}_{j=2} K\bigl(t_{j-2}, x^{j-2} \bigr) \int ^{t_{j+1}}_{t_{j}}(t_{n+1}-\tau )^{\alpha _{1}-1}\,d \tau \\ &{}+\frac{\alpha _{1}}{C(\alpha _{1})\varGamma (\alpha _{1})}\sum^{n}_{j=2} \frac{K(t_{j-1}, x^{j-1} )-K(t_{j-2}, x^{j-2})}{\Delta t} \int ^{t_{j+1}}_{t_{j}}(\tau -t_{j-2}) (t_{n+1}- \tau )^{\alpha _{1}-1}\,d\tau \\ & {}+\frac{\alpha _{1}}{C(\alpha _{1})\varGamma (\alpha _{1})}\sum^{n}_{j=2} \frac{K(t_{j}, x^{j} )-2 K(t_{j-1}, x^{j-1} )+K(t_{j-2}, x^{j-2} )}{2 (\Delta t)^{2}} \\ &{}\times \int ^{t_{j+1}}_{t_{j}}(\tau -{t_{j-2}}) (\tau -{t_{j-1}}) \times (t_{n+1}-\tau )^{\alpha _{1}-1}\,d\tau . \end{aligned}$$

Now, calculating the integrals in equation (23), we obtain the following:

24 $$ \begin{gathered} \int ^{t_{j+1}}_{t_{j}}(t_{n+1}-\tau )^{\alpha _{1}-1}\,d\tau =\frac{(\Delta t)^{\alpha _{1}}}{\alpha _{1}} \bigl[(n-j+1)^{\alpha _{1}}-(n-j)^{\alpha _{1}} \bigr], \\ \int ^{t_{j+1}}_{t_{j}}(\tau -t_{j-2}) (t_{n+1}-\tau )^{\alpha _{1}-1}\,d \tau =\frac{(\Delta t)^{\alpha _{1}+1}}{\alpha _{1}(\alpha _{1}+1)} \bigl[(n-j+1)^{\alpha _{1}}(n-j+3+2 \alpha _{1}) \\ \hphantom{\int ^{t_{j+1}}_{t_{j}}(\tau -t_{j-2}) (t_{n+1}-\tau )^{\alpha _{1}-1}\,d \tau =} {}-(n-j+1)^{\alpha _{1}}(n-j+3+3\alpha _{1}) \bigr], \\ \int ^{t_{j+1}}_{t_{j}}(\tau -{t_{j-2}}) (\tau -{t_{j-1}})\times (t_{n+1}- \tau )^{\alpha _{1}-1}\,d\tau\\ \quad =\frac{(\Delta t)^{\alpha _{1}+2}}{\alpha _{1}(\alpha _{1}+1)(\alpha _{1}+2)} \\ \qquad {} \times \bigl[(n-j+1)^{\alpha _{1}} \bigl[2(n-j)^{2}+(3\alpha _{1}+10) (n-j) +2\alpha ^{2}_{1}+9\alpha _{1}+12 \bigr] \\ \qquad {}-(n-j)^{\alpha _{1}} \bigl[2 (n-j)^{2}+(5 \alpha _{1}+10) (n-j) +6\alpha ^{2}_{1}+18\alpha _{1}+12 \bigr] \bigr], \end{gathered} $$

and inserting them into (23), we get

25 $$\begin{aligned} x^{n+1} =&x^{0}+\frac{1-\alpha _{1}}{C(\alpha _{1})}K \bigl(t_{n}, x(t_{n})\bigr) \\ &{}+ \frac{\alpha _{1} (\Delta t)^{\alpha _{1}}}{C(\alpha _{1})\varGamma (\alpha _{1}+1)} \sum^{n}_{j=2} K\bigl(t_{j-2}, x^{j-2} \bigr) \bigl[(n-j+1)^{\alpha _{1}}-(n-j)^{ \alpha _{1}} \bigr] \\ &{}+ \frac{\alpha _{1} (\Delta t)^{\alpha _{1}}}{C(\alpha _{1})\varGamma (\alpha _{1}+2)} \sum^{n}_{j=2} \bigl[K\bigl(t_{j-1}, x^{j-1} \bigr)-K\bigl(t_{j-2} x^{j-2}\bigr) \bigr] \\ &{}\times \bigl[(n-j+1)^{\alpha _{1}}(n-j+3+2 \alpha _{1})-(n-j+1)^{ \alpha _{1}}(n-j+3+3 \alpha _{1}) \bigr] \\ & {}+ \frac{\alpha _{1} (\Delta t)^{\alpha _{1}}}{2 C(\alpha _{1})\varGamma (\alpha _{1}+3)}\sum^{n}_{j=2} \bigl[K\bigl(t_{j}, x^{j} \bigr)-2 K \bigl(t_{j-1}, x^{j-1} \bigr)+K\bigl(t_{j-2}, x^{j-2} \bigr) \bigr] \\ &{} \times \bigl\{ (n-j+1)^{\alpha _{1}} \bigl[2(n-j)^{2}+(3\alpha _{1}+10) (n-j)+2 \alpha ^{2}_{1}+9\alpha _{1}+12 \bigr] \\ &{}-(n-j)^{\alpha _{1}} \bigl[2 (n-j)^{2}+(5 \alpha _{1}+10) (n-j)+6 \alpha ^{2}_{1}+18\alpha _{1}+12 \bigr] \bigr\} . \end{aligned}$$

Finally, we have the following approximation:

26 $$\begin{aligned} x^{n+1} =&x^{0}+\frac{1-\alpha _{1}}{C(\alpha _{1})} \alpha _{2} t^{ \alpha _{2}-1}_{n} K \bigl(t_{n}, x(t_{n})\bigr) \\ &{}+ \frac{\alpha _{1}\alpha _{2} (\Delta t)^{\alpha _{1}}}{C(\alpha _{1})\varGamma (\alpha _{1}+1)}\sum^{n}_{j=2} t^{\alpha _{2}-1}_{j-2} K\bigl(t_{j-2}, x^{j-2} \bigr) \bigl[(n-j+1)^{\alpha _{1}}-(n-j)^{ \alpha _{1}} \bigr] \\ &{}+ \frac{\alpha _{1} \alpha _{2}(\Delta t)^{\alpha _{1}}}{C(\alpha _{1})\varGamma (\alpha _{1}+2)} \sum^{n}_{j=2} \bigl[t^{\alpha _{2}-1}_{j-1}K\bigl(t_{j-1}, x^{j-1} \bigr)-t^{ \alpha _{2}-1}_{j-2}K \bigl(t_{j-2}, x^{j-2}\bigr) \bigr] \\ &{}\times \bigl[(n-j+1)^{\alpha _{1}}(n-j+3+2 \alpha _{1})-(n-j+1)^{ \alpha _{1}}(n-j+3+3 \alpha _{1}) \bigr] \\ &{} + \frac{\alpha _{1} \alpha _{2}(\Delta t)^{\alpha _{1}}}{2 C(\alpha _{1})\varGamma (\alpha _{1}+3)}\sum^{n}_{j=2} \bigl[t^{\alpha _{2}-1}_{j}K\bigl(t_{j}, x^{j} \bigr)-2 t^{\alpha _{2}-1}_{j-1}K \bigl(t_{j-1}, x^{j-1} \bigr)+t^{\alpha _{2}-1}_{j-2}K \bigl(t_{j-2}, x^{j-2} \bigr) \bigr] \\ &{} \times \bigl\{ (n-j+1)^{\alpha _{1}} \bigl[2(n-j)^{2}+(3\alpha _{1}+10) (n-j)+2 \alpha ^{2}_{1}+9\alpha _{1}+12 \bigr] \\ &{}-(n-j)^{\alpha _{1}} \bigl[2 (n-j)^{2}+(5 \alpha _{1}+10) (n-j)+6 \alpha ^{2}_{1}+18\alpha _{1}+12 \bigr] \bigr\} . \end{aligned}$$

## Estimation of parameters

**Figure 2:**
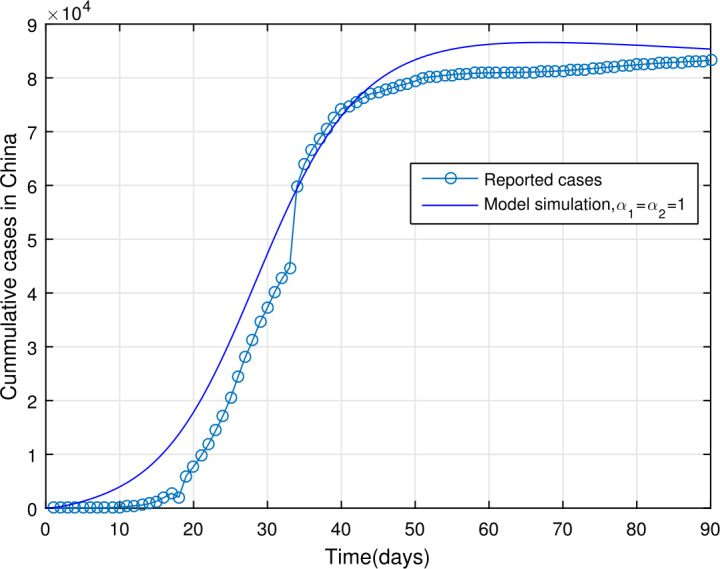
Reported number of COVID-19 cases in China versus model fit, $\alpha _{1}=\alpha _{2}=1$

**Figure 3:**
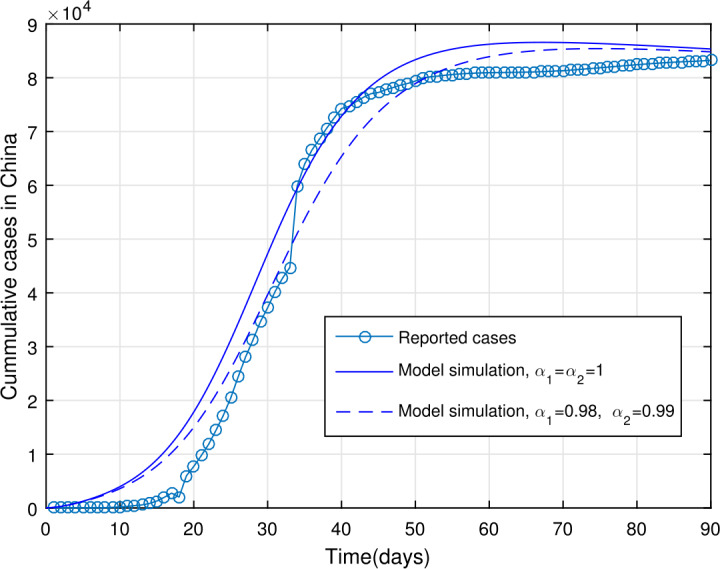
Reported number of COVID-19 cases in China versus model fit, $\alpha _{1}=0.99$, $\alpha _{2}=0.98$

In order to obtain the model parameters based on the real data of COVID-19 of the mainland China, we consider some of the parameters such as the birth and death rates from the literature while the rest of the parameters have been fitted to the data. We consider the data of WHO [28] from January 11, 2020 until April 9, 2020, with total reported daily cases being 83249 with 3344 deaths. For parameterizations of model (7), we fixed $\alpha _{1}=\alpha _{2}=1$ and simulated the model using the least-squares fitting; the obtained realistic parameters are as shown in Table 1. The total population of China is considered to be 1,300,000,000, with $N(0)=1\mbox{,}300\mbox{,}000\mbox{,}000$. The cumulative number of cases suggests that the initial value of the infected individuals is $I(0)=41$, with the possible exposed cases due to fitting being $E(0)=20\mbox{,}000$. The susceptible population in the absence of disease is estimated to be $S(0)=1\mbox{,}299\mbox{,}979\mbox{,}959$ while the other compartments of the model with the initial conditions are considered to be $A(0)=0$, $Q(0)=0$, $H(0)=0$, $R(0)=0$, and $M(0)=44\mbox{,}000$ (subject to data fitting). The birth rate is calculated as $\varLambda =46\mbox{,}381$*per day*, while the natural death rate is given by $\mu =1/76.79$*per day*. The estimated basic reproduction number for the mainland China for the given period of infected cases is obtained as $\mathcal{R}_{0}\approx 6.6361$. The parameter values in Table 1 are used to show the model (7) versus data fitting in Figs. 2 and 3. In Fig. 2, we show the model fitting versus data when $\alpha _{1}=\alpha _{2}=1$ while Fig. 3 is plotted in order to show the effectiveness of the fractal-fractional model when $\alpha _{1}=0.99$, $\alpha _{2}=0.98$. The result in Fig. 3 is better than that with integer order derivative.

**Table 1:** The estimated and fitted parameter values for model (7), when $\alpha _{1}=\alpha _{2}=1$

Parameter	Description	Value	Source
*Λ*	Birth rate	*μ* × *N*(0)	Estimated
*μ*	Natural death rate	$\frac{1}{76.79\times 365}$	[29]
$\eta _{1}$	Contact rate	0.003	Fitted
$\eta _{2}$	Disease transmission coefficient	0.00000034002	Fitted
*ψ*	Transmissibility multiple	0.004	Fitted
*θ*	Asymptomatic infection	0.21003	Fitted
*ω*	Incubation period	0.00001111	Fitted
*ρ*	Incubation period	0.0180322	Fitted
$\tau _{1}$	Recovery rate due to *I*	0.00023	Fitted
$\tau _{2} $	Recovery rate due to *A*	0.19	Fitted
$q_{1}$	Infection contribution to *M* by *I*	0.00101	Fitted
$q_{1}$	Infection contribution to *M* by *A*	0.0214	Fitted
$q_{3} $	Removing rate of virus from *M*	0.23008	Fitted
$\delta _{1}$	Quarantine rate of exposed individuals	0.1223	Fitted
$\xi _{1}$	Disease death rate of infected individuals *I*	0.0002	Fitted
*γ*	Hospitalization rate of infected individuals	0.0005	Fitted
$\phi _{1}$	Recovery rate of quarantined individuals	0.1	Fitted
$\delta _{2}$	Hospitalization rate of quarantined individuals	0.06	Fitted
$\phi _{2}$	Recovery rate of hospitalized individuals	0.2	Fitted
$\xi _{2}$	Disease death rate of hospitalized individuals	0.01	Fitted

## Numerical results

**Figure 4:**
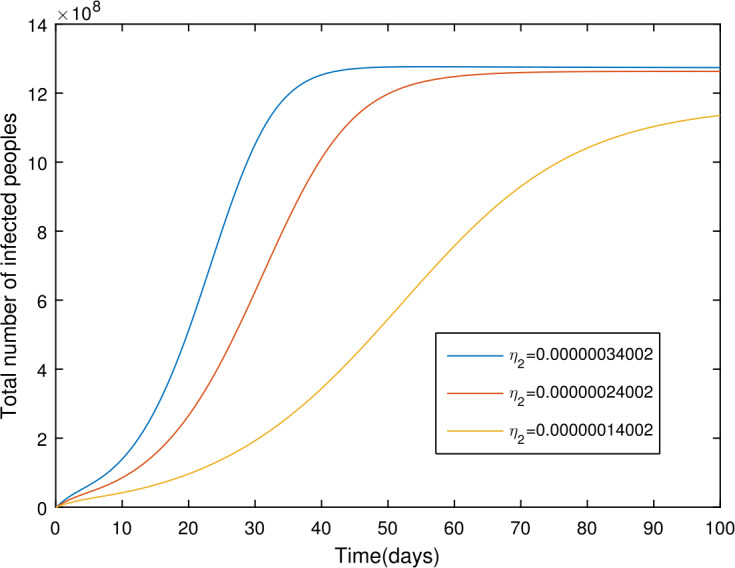
The total number of infected people for various values of $\eta _{2}$

**Figure 5:**
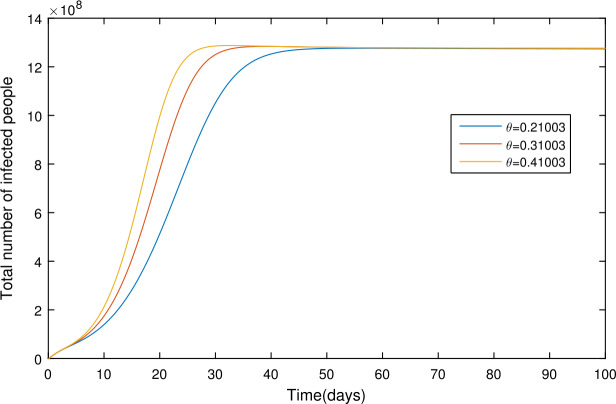
The total number of infected people for various values of *θ*

**Figure 6:**
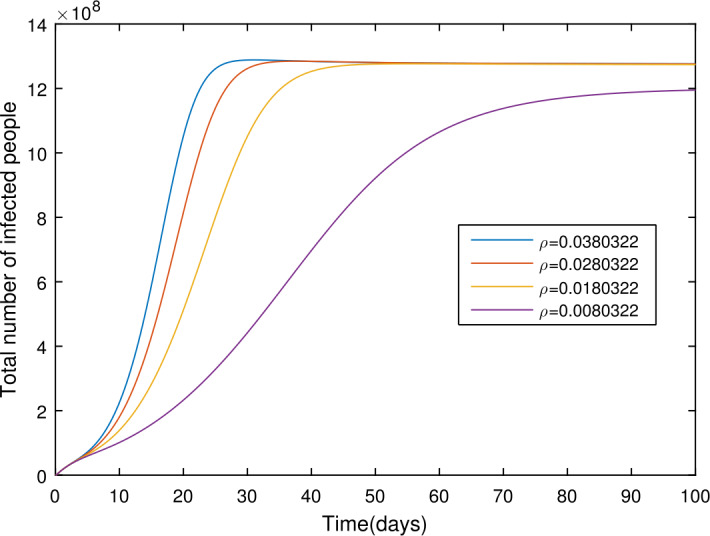
The total number of infected people for various values of *ρ*

**Figure 7:**
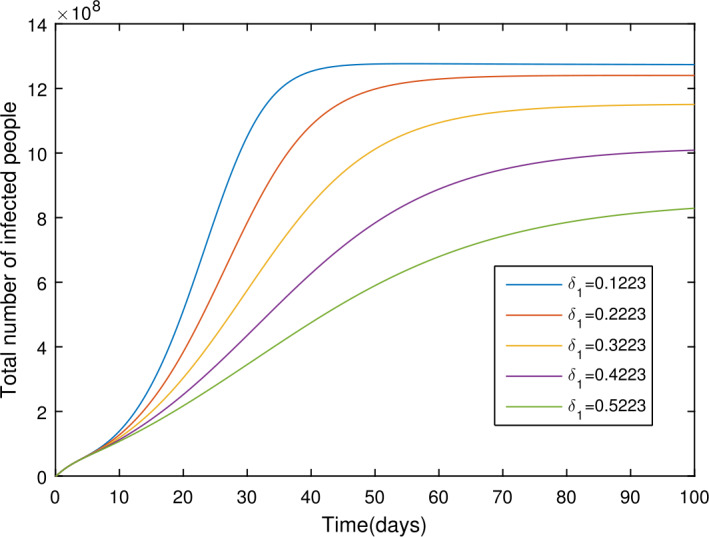
The total number of infected people for various values of $\delta _{1}$

**Figure 8:**
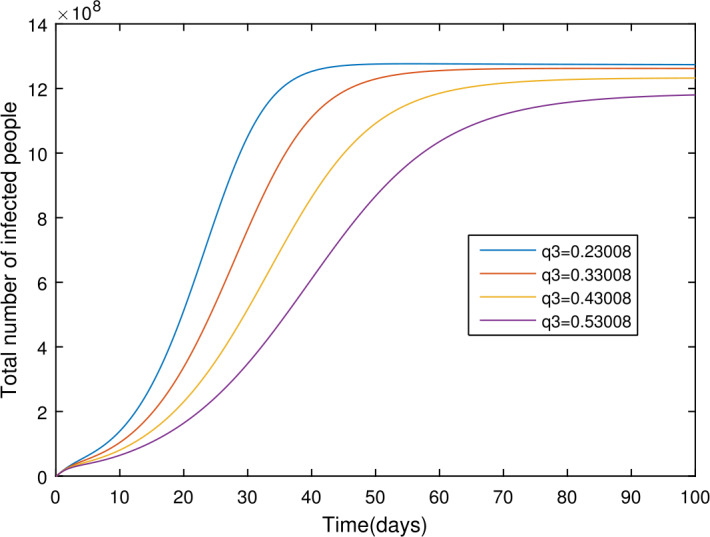
The total number of infected people for different values of $q_{3}$

**Figure 9:**
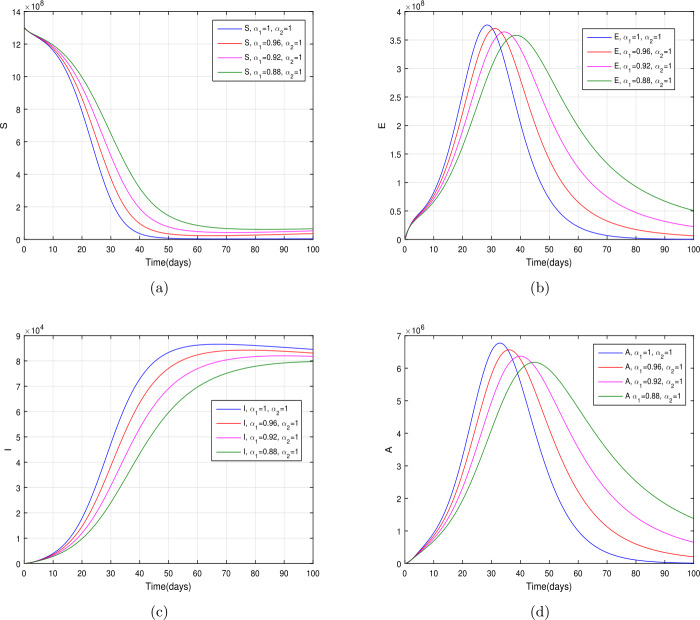
The dynamics of the model variables for $\alpha _{1}=1, 0.96, 0.92, 0.88$ and $\alpha _{2}=1$, subfigures (a)–(d) respectively represent the susceptible, exposed, infected, and asymptomatic individuals

**Figure 10:**
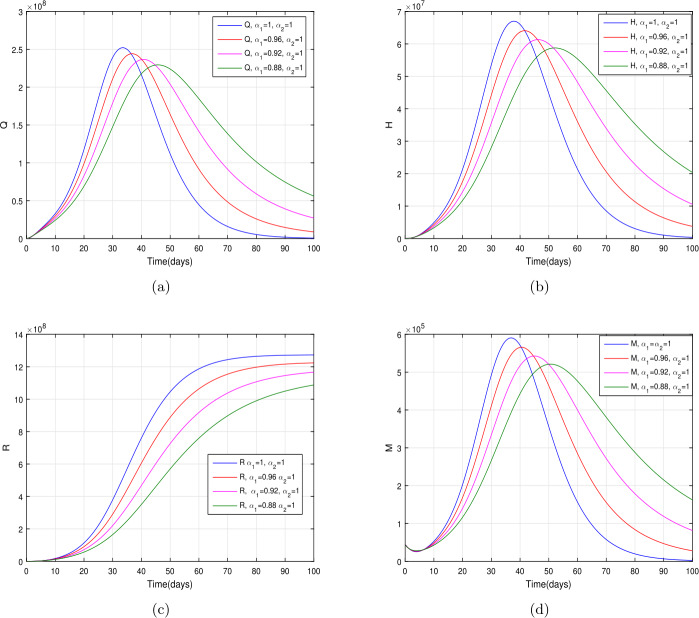
The dynamics of the model variables for $\alpha _{1}=1, 0.96, 0.92, 0.88$ and $\alpha _{2}=1$, subfigures (a)–(d) respectively represent the quarantined, hospitalized, recovered, and contaminated environment

**Figure 11:**
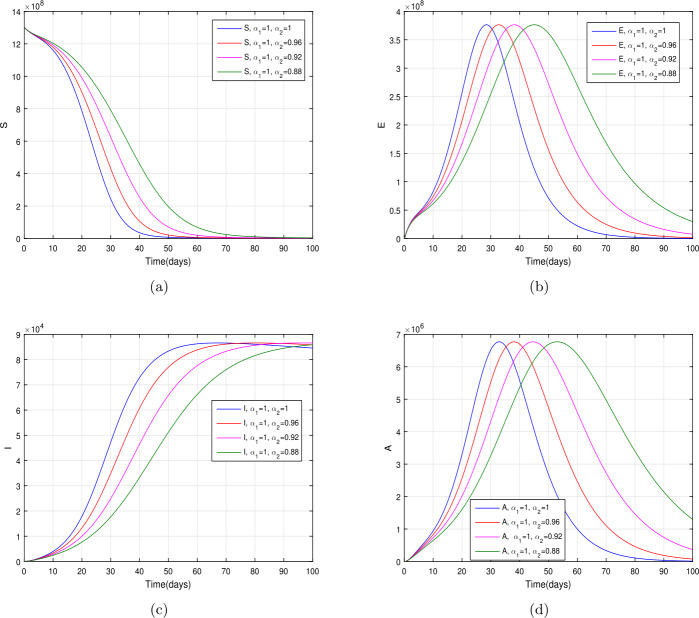
The dynamics of the model variables for $\alpha _{2}=1, 0.96, 0.92, 0.88$ and $\alpha _{1}=1$, subfigures (a)–(d) respectively represent the susceptible, exposed, infected, and asymptomatic individuals

**Figure 12:**
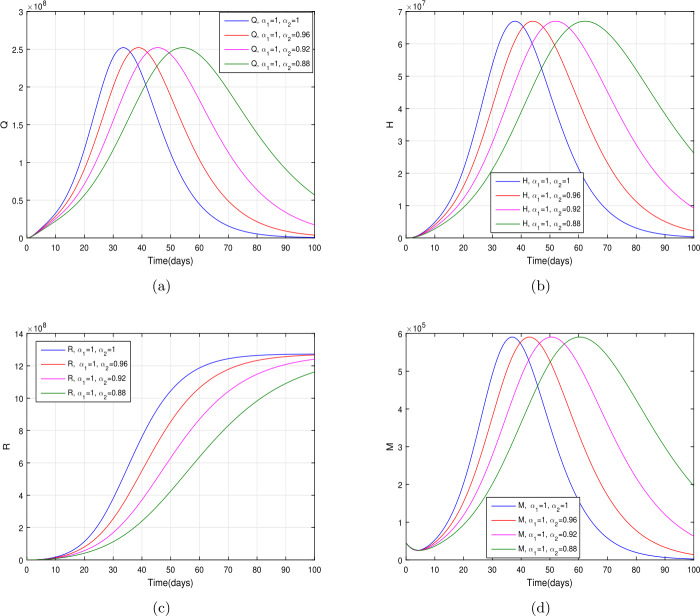
The dynamics of the model variables for $\alpha _{2}=1, 0.96, 0.92, 0.88$ and $\alpha _{1}=1$, subfigures (a)–(d) respectively represent the quarantined, hospitalized, recovered, and contaminated environment

**Figure 13:**
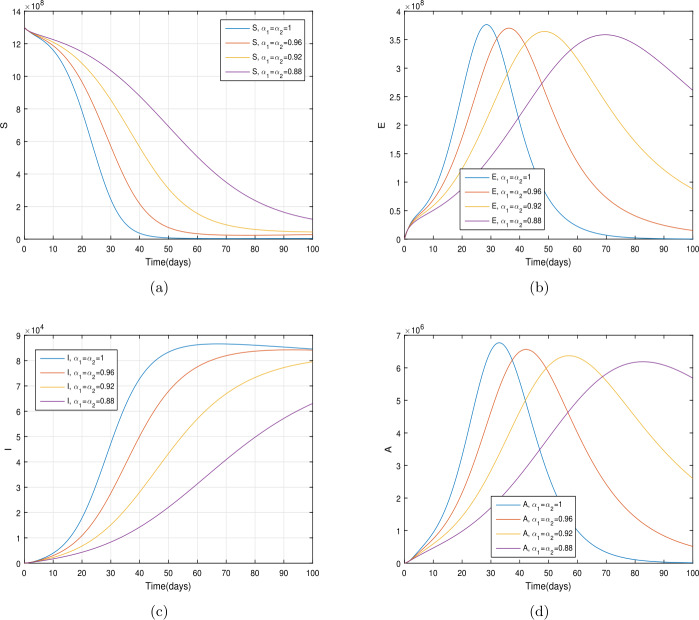
The dynamics of the model variables for $\alpha _{1}=\alpha _{2}=1, 0.96, 0.92, 0.88$, subfigures (a)–(d) respectively represent the susceptible, exposed, infected, and asymptomatic individuals

**Figure 14:**
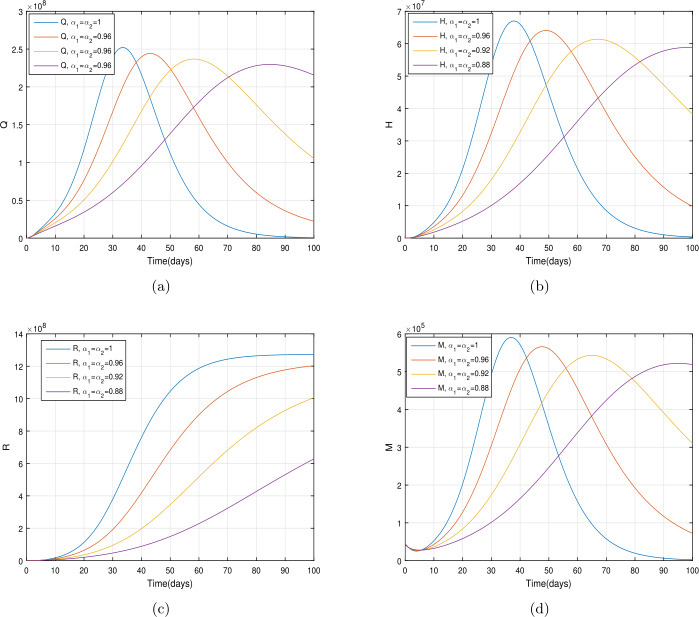
The dynamics of the model variables for $\alpha _{1}=\alpha _{2}=1, 0.96, 0.92, 0.88$, subfigures (a)–(d) respectively represent the quarantined, hospitalized, recovered, and contaminated environment

In the present section, we are studying model (7) numerically by using the novel approach presented above. We consider the unit of time being *a day*. The parameter values considered in this simulation are shown in Table 1. Figures 2 and 3 show the curve fitting with integer and noninteger order. The graphical results show the importance of the fractal-fractional operator for data comparison. The total number of infected people for different values of parameter $\eta _{2}$ is shown. Decreasing the infection in the seafood market, reduces the number of total infected decreases very fast, see Fig. 4. Thus, the closing of the seafood market by the Chinese government was an important decision to control the spread of the infection further. The proportion of asymptomatic infection parameter *θ* is shown graphically in Fig. 5. By decreasing the value of *θ*, the total number of infected people is decreasing. Therefore, the asymptomatic infection plays an important role in the infection generation, and therefore, the people should be educated to avoid the interaction with such people. Similarly, the effect of parameters *ρ*, $\delta _{1}$, and $q_{3}$ are shown in Figs. 6–8. Also, by decreasing the values of these parameters, the total number of infected people is decreasing. Therefore, the quarantine class is important in the modeling of novel coronavirus. In Figs. 9 to 14, we present the dynamics of the model variables for fractal and fractional order parameter values. In Figs. 9 and 10, we choose $\alpha _{2}=1$ and $\alpha _{1}=1, 0.96, 0.92, 0.88$. In Figs. 11 and 12, we choose $\alpha _{1}=1$ and $\alpha _{2}=1, 0.96, 0.92, 0.88$. In Figs. 13 and 14, we choose $\alpha _{1}=\alpha _{2}=1, 0.96, 0.92, 0.88$. In these figures with different values of the fractal-fractional operators, a novel analysis and a variety of choices for choosing the fractal and fractional order parameters is extensively illustrated, which is the beauty of the fractal-fractional operator. One can see that the modeling of a real-life problem with fractal-fractional operator is more useful than that with the ordinary derivative. The infected data and its comparison with proposed model and the possible elimination of the infection can be assessed well with this new fractal-fractional operator. Our results suggest that, when decreasing the values of both the fractal and fractional order, one can see a decrease in the infected compartment, which is better than for the integer-order compartment. The suggested fractal and fractional order values are arbitrary, and one can choose any value to simulate the model.

## Conclusions

We investigated the dynamics of COVID-19 with quarantine and isolations with real statistical cases reported in the mainland China. We first developed the model using ordinary derivative and then used the fractal-fractional derivative in Atangana–Baleanu sense to generalize the model. The mathematical results for the model were shown. The stability of the model for disease-free case is obtained for $\mathcal{R}_{0}<1$. We use the real cases in the mainland of China for the model parameterizations. Using the realistic parameter values, we obtained the basic reproduction number $\mathcal{R}_{0}\approx 6.6361$. We considered a new numerical technique, which is very accurate for the solution of fractional differential equations, and obtained results for the proposed model. The curve fitting for the integer and noninteger cases has been shown and proved that the fractal-fractional model is more suitable than the classical one. We consider many parameters and their effect on the model graphically, which can be regarded as the controls for disease eradication. The fractal-fractional model was used further to simulate it and obtained many graphical results for various values of the fractal and fractional orders. We considered some of the key parameters as controls with suggested values to obtain the possible elimination of the disease in the society. The biological explanation of the key parameters, such as *ρ*, $\delta _{1}$, etc., has been already explained in the model formulation section in details. The results in the paper are very useful in the early eradication of the disease in the community. In the future, this model can be extended by using other fractional operators and numerical schemes to obtain new and more results about the dynamics of COVID-19. Further, the effect of saturated incidence rate can also be considered to extend this model and obtain the results.
